# Reduced ability to neutralize the Omicron variant among adults after infection and complete vaccination with BNT162b2, ChAdOx1, or CoronaVac and heterologous boosting

**DOI:** 10.1038/s41598-023-34035-9

**Published:** 2023-05-08

**Authors:** Otávio Melo Espíndola, Trevon L. Fuller, Mia Ferreira de Araújo, Luis Fernando Lopez Tort, Lusiele Guaraldo, Guilherme Calvet, Paola Resende, Myrna Bonaldo, Jimmy Whitworth, Chris Smith, Marilda Siqueira, Patrícia Brasil

**Affiliations:** 1 Evandro Chagas National Institute of Infectious Diseases, Oswaldo Cruz Foundation (Fiocruz), Avenida Brasil 4365, Rio de Janeiro, RJ 21040-900 Brazil; 2 Institute of the Environment and Sustainability, University of California Los Angeles, Los Angeles, 90095 USA; 3 Laboratory of Respiratory Viruses and Measles National Influenza Centre (GISRS-WHO)-Americas Regional Reference Lab for Measles and Rubella-Reference Laboratory for COVID-19 (WHO), Oswaldo Cruz Institute (IOC), Fiocruz, Rio de Janeiro, 21040-900 Brazil; 4 Laboratory of Molecular Virology, Biological Sciences Department, Universidad de la República, Salto, Uruguay; 5 Laboratory of Molecular Biology, Oswaldo Cruz Institute, Fiocruz, Rio de Janeiro, 21040-900 Brazil; 6 Department of Clinical Research, London School of Hygiene and Tropical Medicine, London, WC1E 7HT UK

**Keywords:** Antibodies, Viral infection, SARS-CoV-2

## Abstract

COVID-19 vaccines have dramatically reduced rates of severe infection requiring hospitalization. However, SARS-CoV-2 variants have reduced vaccine effectiveness at preventing any symptomatic infection. This real-world study analyzed binding and neutralizing antibodies generated after complete vaccination and boosting across three vaccine platforms. Binding antibodies decayed most slowly in people under 60 with hybrid immunity. Neutralizing antibodies against Omicron BA.1 were reduced compared to other variants. The anamnestic anti-spike IgG response to the first boost was more pronounced than after the second boost. Monitoring of the effects of SARS-CoV-2 mutations on disease severity and the effectiveness of therapeutics is warranted.

## Introduction

The introduction of vaccination against Coronavirus disease 2019 (COVID-19) in December 2020 has dramatically reduced the pressure on health services worldwide by preventing morbidity, hospitalizations, and death^1^. Initially, immunization strategies relied mainly on the availability of vaccines approved by government agencies after controlled clinical trials. Most approved COVID-19 vaccines fall into three groups: inactivated virus, adenoviral vector, and messenger RNA (mRNA). Such vaccines have proven safe, but differences are observed in their efficacy in preventing symptomatic COVID-19, with mRNA vaccines such as BNT162b2 (Pfizer-BioNTech) and mRNA-1273 (Moderna) exhibiting the highest vaccine efficacy, usually over 90%^2^. In turn, adenovirus-based vaccines, such as ChAdOx1 (AstraZeneca) and the single-dose Ad26.COV2.S (Janssen) and inactivated virus vaccines, such as CoronaVac (Sinovac Biotech), showed intermediate protection rates of around 70%^2^.

Low adherence to social distancing measures, unequal global distribution of vaccines, and low vaccination rates have favored SARS-CoV-2 dissemination and the emergence of virus variants. From September 2020 to 2021, several variants of concern (VoCs) emerged, exhibiting mutations in the spike protein and displaying a high potential for transmission and immune evasion. The main VoCs identified to date have been Alpha (B.1.1.7), Beta (B.1.351), Gamma (P.1/P.1.*), Delta (B.1.617.2/AY.*), and Omicron (B.1.1.529/BA.*). Furthermore, many Omicron lineages have been identified throughout 2022, including BA.1, BA.1.1, BA.2, BA.2.75, BA.3, BA.4, BA4.6, BA.5, and BQ.1, and BQ.1.1, which show common antigenic characteristics^3^.

As in many other countries, Brazil’s COVID-19 immunization program implemented heterologous vaccination. There was widespread use of CoronaVac or ChAdOx1 for the first two doses followed by BNT162b2 or ChAdOx1 for boosting and very few people received BNT162b2 as the first dose. The rapid dissemination of the Omicron VoC has generated interest in the duration of immunity induced only by complete vaccination (two doses) or followed by a viral infection, referred to as hybrid immunity. In both cases, individuals may have benefited from a booster (third dose), restoring high anti-spike IgG levels^4^.

In contrast, it is unclear whether individuals benefit from a second booster (fourth dose), given the significant antigenic divergences between available vaccines and Omicron variants. This work evaluates the magnitude and dynamics of anti-spike IgG serum levels after a complete vaccination with different vaccine platforms. We determined the impact of hybrid immunity on anti-spike IgG decay. We also performed SARS-CoV-2 serum neutralization assays utilizing Zeta, Gamma, Delta, and Omicron VoCs to define the extent of viral escape.

## Results

The median age of the study participants was 44 (IQR 33–59) (Table 1). Fully 68.7% (N = 360) of the participants were women. The most frequent level of educational attainment was graduated from secondary school but did not graduate from college (N = 217, 41.4%). The most frequent categories of race/ethnicity were white (N = 232, 44.3%) and multiracial (N = 172, 32.8%).

**Table 1:** Demographic characteristics and educational attainment of study participants.

Characteristics	Frequency (%)
Age (years)	
≤ 20, N (%)	31 (5.9%)
21–30, N (%)	127 (24.2%)
31–40, N (%)	73 (13.9%)
41–50, N (%)	100 (19.1%)
51–60, N (%)	80 (15.3%)
Over 60 years of age, N (%)	114 (21.8%)
Female sex, N (%)	360 (68.7%)
Education	
Illiterate or did not graduate from primary school, N (%)	69 (13.2%)
Graduated from primary school but did not graduate from secondary school, N (%)	87(16.6%)
Graduated from secondary school but did not graduate from college, N (%)	217 (41.4%)
Graduated from college, N (%)	129 (24.6%)
Education missing, N (%)	22 (4.2%)
Race/ethnicity	
Asian	2 (0.4%)
Black	97 (18.5%)
Multiracial	172 (32.8%)
White	232 (44.3%)
Ethnicity/race missing	21 (4%)

**Figure 1:**
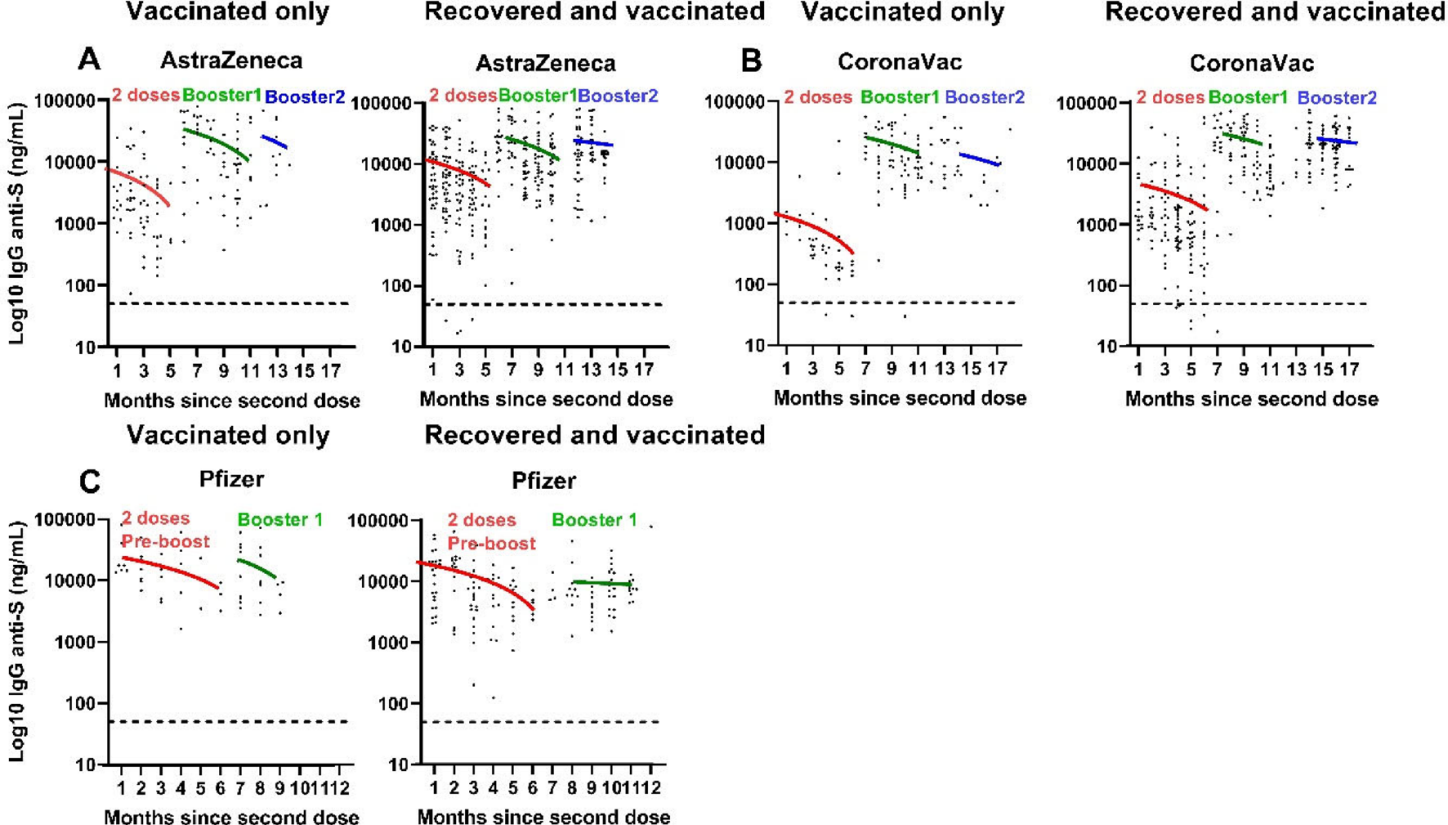
Dynamics of anti-spike IgG responses after full vaccination and boosting. Serum samples were monthly collected and evaluated for anti-spike IgG levels by chemiluminescent assay after the full immunization scheme (two doses) with (**A**) ChAdOx1 (AstraZeneca) (n = 495), (**B**) CoronaVac (Sinovac Biotech) (n = 472), and (**C**) BNT162b2 (Pfizer-BioNTech) (n = 201), and after the first and second heterologous boosting carried out with ChAdOx1 or BNT162b2. The dynamics of anti-spike decay was evaluated considering the immunity against COVID-19 induced by vaccination only and by vaccination and infection (recovered and vaccinated).

Consecutive serum samples (n = 1168), corresponding to 524 individuals, were collected after every vaccination and/or infection. Samples from individuals fully immunized with ChAdOx1 (n = 495) and BNT162b2 (n = 201) (Fig. 1A,C) had higher levels of anti-spike IgG than those that had received CoronaVac (n = 472) (Fig. 1B). After the first boost, which was carried out with BNT162b2 or ChAdOx1, the fold rise in IgG titers was 20.96 (95% CI 10.6–41.4) for CoronaVac, 7.08 (CI 6.9–7.3) for ChAdOx1, and 1.68 (CI 1.4–1.9) for BNT162b2. After the second boost, the fold rise was 1.09 (CI 0.18–6.62) for CoronaVac and 0.77 (0.23–2.54) for ChAdOx1. Individuals who received a second booster were similar in age to the rest of the study participants (*p* = 0.17), but were more likely to be female (*p* = 0.001) (Supplementary Table S1). There were too few second booster doses to calculate the fold change for BNT162b2.

**Figure 2:**
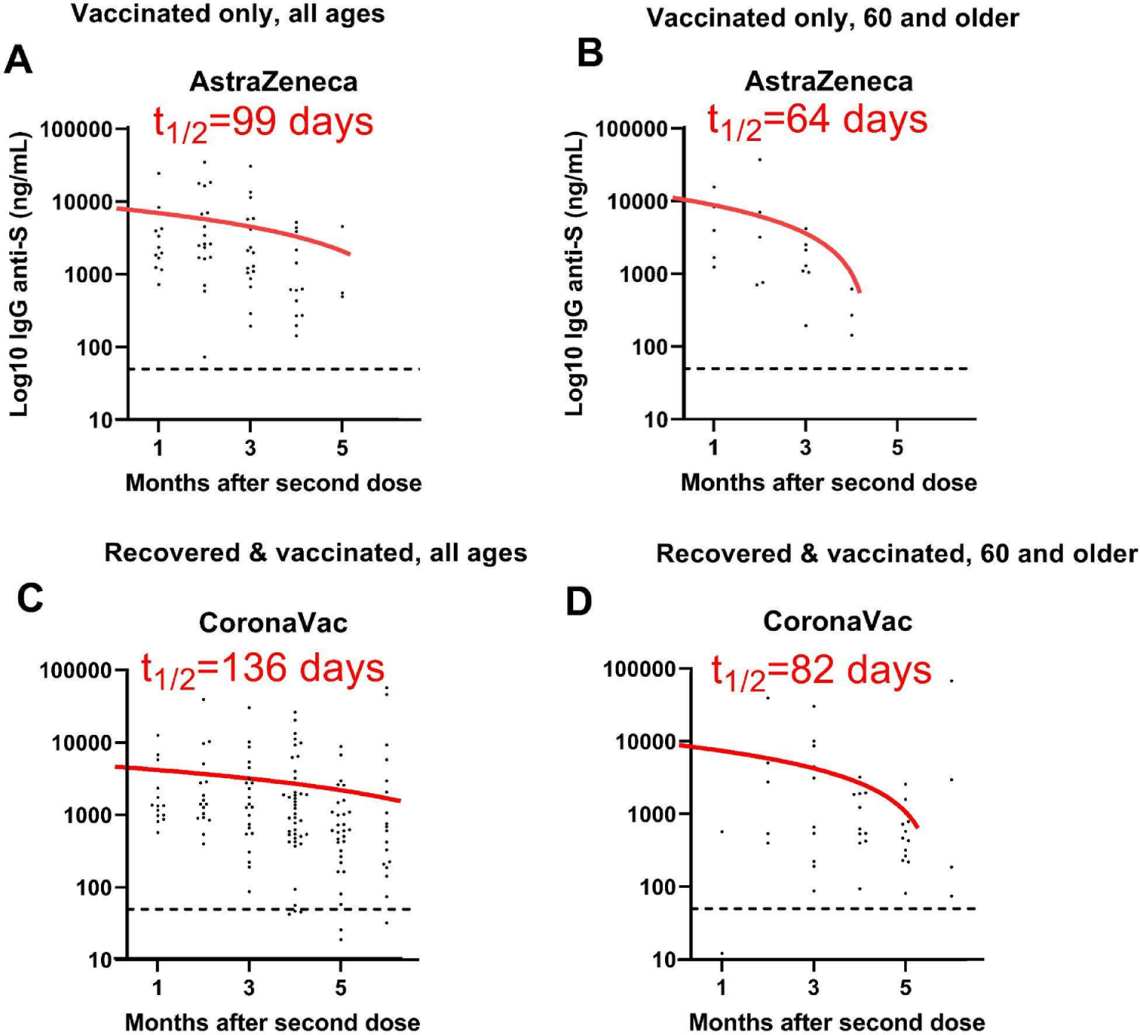
Dynamics of anti-spike IgG responses after full vaccination according to the age. Serum samples were monthly collected and evaluated for anti-spike IgG levels by chemiluminescent assay after the second vaccine dose in (**A**,**B**) participants comparing all age groups to individuals of 60 years and older for AstraZeneca (ChAdOx1) vaccinated only, and for (**C**,**D**) CoronaVac vaccinated and recovered from infection. Half-life estimates were based on a linear model of antibody waning.

**Figure 3:**
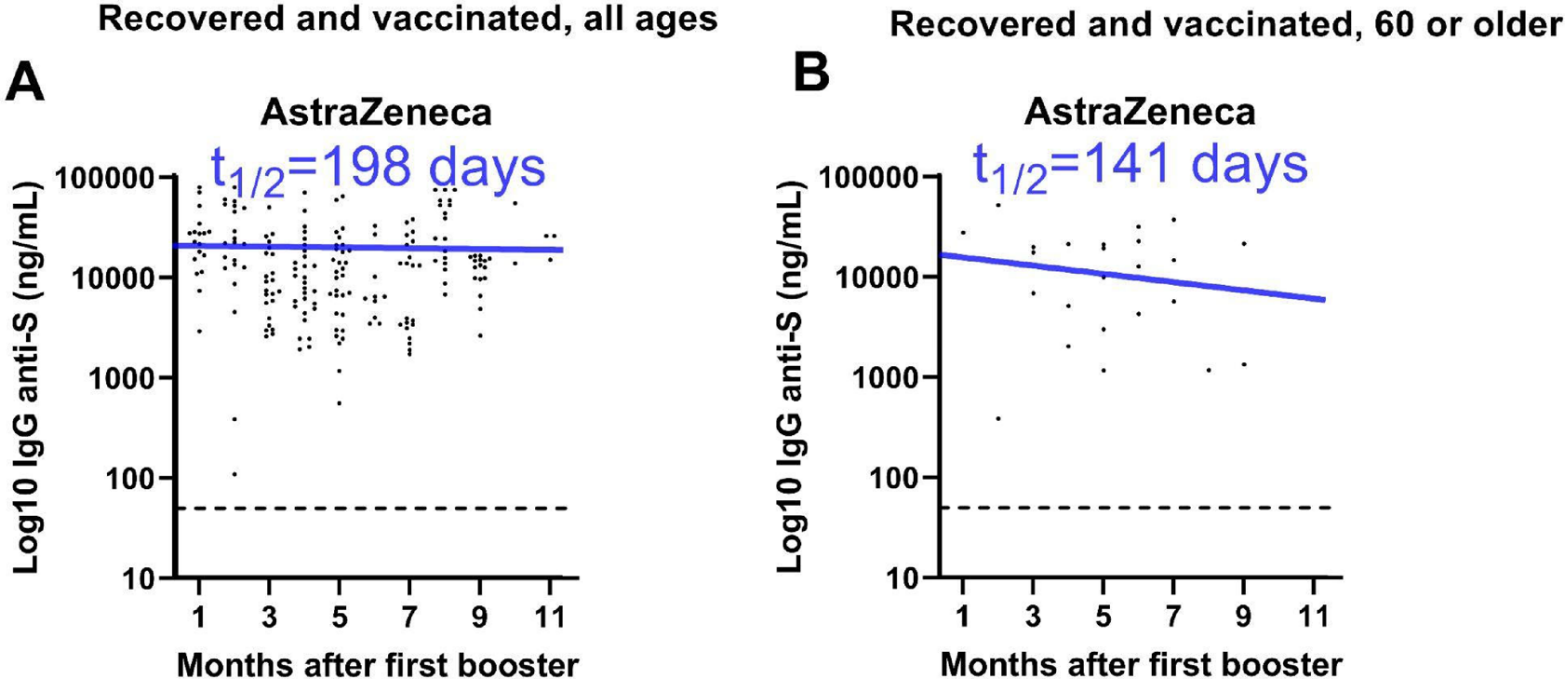
Dynamics of anti-spike IgG responses after full vaccination with AstraZeneca (ChAdOx1) according to age and history of infection. Serum samples were monthly collected and evaluated for anti-spike IgG levels by chemiluminescent assay after the first booster dose in participants that were recovered from infection comparing (**A**) all age groups to (**B**) individuals of 60 years and older.

Anti-spike IgG was also evaluated against the background of SARS-CoV-2 infection. Vaccinated-only and vaccinated/infected individuals presented similar anti-spike IgG levels (Fig. 1), although the latter group displayed a slower antibody decay, independently of the vaccine type (Fig. 2). To estimate the impact of immunosenescence on the duration of anti-SARS-CoV-2 humoral response, the half-life of anti-spike IgG levels was calculated for individuals of all ages and those over 60. Vaccinees over 60 had faster antibody decay compared to data from all study participants (Figs. 2, 3). Anti-spike IgG half-life had a 30% reduction after full immunization with ChAdOx1, dropping from 99 days for all participants to 64 days for those over 60. Individuals with hybrid immunity had slower antibody decay, regardless of age and vaccine type. The analysis including all participants infected before immunization revealed anti-spike IgG half-lives of 198 and 136 days for ChAdOx1 and CoronaVac, respectively (Figs. 2, 3). In contrast, people over 60 also had a shorter anti-spike IgG half-life when vaccinated with ChAdOx1 (141 days) or CoronaVac (82 days) (Figs. 2, 3).

### Virus neutralization testing

**Figure 4:**
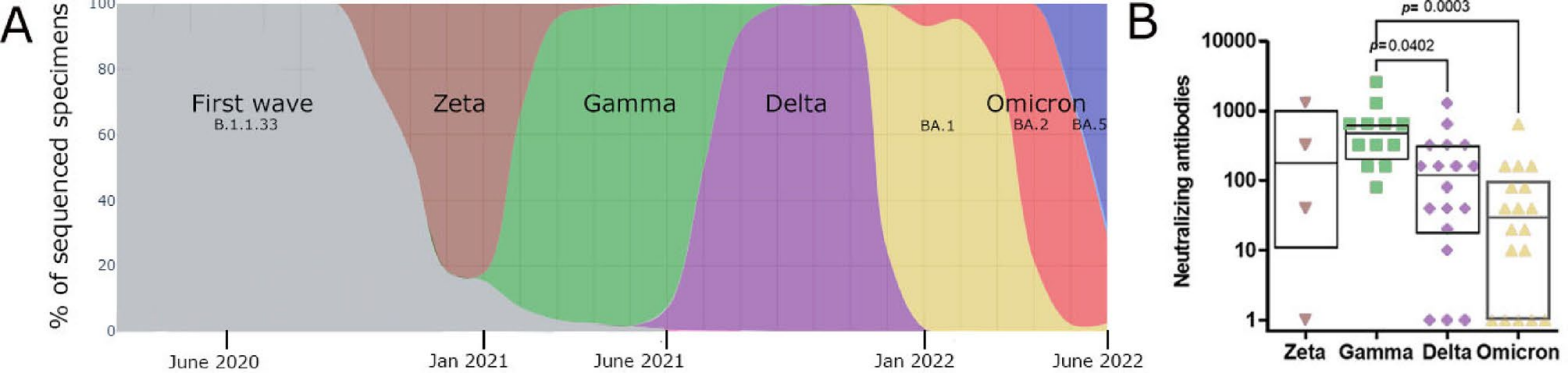
SARS-CoV-2 neutralization by serum samples from individuals with hybrid immunity. (**A**) Timeline of SARS-CoV-2 variants of concern (VoCs) circulating in Brazil along the recruitment of study participants. (**B**) Serum samples from individuals vaccinated and infected before the emergence of Omicron VoCs were assessed to determine the titers of neutralizing antibodies against Zeta (P.2), Gamma (P.1), Delta (B.1.617.2) and Omicron (BA.1) VoCs by 90% plaque reduction neutralization test (PRNT_90_).

The serum from individuals vaccinated/infected before the emergence of Omicron VoCs exhibited high titers of neutralizing antibodies against the VoCs Gamma and Zeta. However, the neutralization of the VoC Delta was two times lower than that of the earlier the VoC Gamma (*p* = 0.0402), and that of Omicron BA.1 was 16 times lower (*p* = 0.0003) (Fig. 4).

## Discussion

In this study, we showed that prior infection was able to prime the immune response to vaccination, independently of the vaccine type, and favored longer humoral immunity than vaccination alone. Although other studies also demonstrated that SARS-CoV-2 infection significantly increased antibody peak level and anti-spike IgG half-life upon vaccination with two doses of ChAdOx1 and BNT162b2^5,6^, there is scant data on the inactivated virus vaccine CoronaVac. We observed that individuals over 60 had estimated serum anti-spike IgG half-life reduced by approximately one-third, suggesting that boosting should therefore prioritize the elderly and clinically vulnerable populations. Immunization of these groups and healthcare workers in Brazil was first carried out with CoronaVac, which showed lower efficacy and reduced anti-spike IgG levels compared with ChAdOx1 and BNT162b2^2^. However, heterologous boosting with ChAdOx1 or BNT162b2 resulted in high anti-spike IgG levels, representing an interesting approach to provide higher protection levels against COVID-19 in countries that extensively used CoronaVac. We also evaluated the effect of the second booster on anti-spike IgG levels. In contrast to the strong recovery of antibody responses after the first boost (third dose), particularly for CoronaVac recipients, a second boost (fourth dose) after six months slightly increased anti-spike IgG due to the high baseline levels. This suggests that the impact on later doses may be minimal, and longer intervals between boosters may be adequate.

The magnitude of neutralizing antibodies and anti-spike IgG responses have been shown as immune correlates of protection for symptomatic SARS-CoV-2 infection^5^. However, the emergence of the Omicron VoC and its lineages has challenged the effectiveness of immunity induced by vaccination and/or natural infection with pre-Omicron SARS-CoV-2 VoCs. Since January 2022, many countries have experienced new waves of COVID-19 cases, including reinfections, associated with new Omicron lineages^3,6,7^. However, increased numbers of cases were not followed by increased hospitalizations and deaths in regions with high vaccination coverage^7^, indicating a reduction in the efficacy of existing vaccines in preventing SARS-CoV-2 transmission but continuing efficacy against severe COVID-19 and death^6^. Our data are in line with these observations since neutralizing antibodies elicited by pre-Omicron VoCs had limited ability to neutralize Omicron BA.1, despite the antibody levels induced by vaccination and/or infection, suggesting that updating COVID-19 vaccines could be beneficial.

In a nation-wide survey performed in the United Kingdom before the emergence of the VoC Omicron lineages, protection rate was estimated to be 67% against SARS-CoV-2 infection for 2–3 months after vaccination with ChAdOx1, and for 5–8 months for BNT162b2, in individuals without prior infection^7^. The effectiveness of BNT162b2 against hospitalization for COVID-19 was reduced from 93 to 70% during the Omicron period^6^. However, individuals immunized by vaccination or natural infection do benefit from partial immunity against Omicron due to antigenic similarity with previous SARS-CoV-2 VoCs, since reinfections have 90% lower odds of resulting in hospitalization or death than primary infections^8^. Antibody and cytotoxic CD8^+^ T-cell responses induced in previously infected individuals or vaccinees might explain this^9,10^.

A shortcoming of the study is the small sample size, which limits the statistical power of our results. Furthermore, number of individuals in our cohort who received BNT162b2 in the initial immunization scheme was low. Therefore, we could not analyze the antibody waning for this vaccine in adults and the elderly. Another potential limitation is that in the analysis of anti-spike IgG most participants who received a second booster of ChAdOx1 or Coronavac were women. This raises the possibility that the data on the dynamics of IgG response after the second booster were biased due to a lack of male participants. However, a large real-world study found no sex differences in the immunogenicity of ChadOx1^11^. In light of this, we believe that the risk of a sex bias in the immunogenicity data is small. In addition, despite the small number of samples tested, it was possible to show significant differences in the titers of neutralizing antibodies according to SARS-CoV-2 VoCs. Thus, our data will help justify future studies on the kinetics of humoral responses against SARS-CoV-2 with larger samples and over a longer period to determine the duration of immunity after infection or vaccination.

In conclusion, our data showed that booster doses should be recommended to restore anti-spike IgG levels in both vaccinated and infected individuals, particularly for CoronaVac vaccinees. However, with the continuing emergence of SARS-CoV-2 VoCs, future immunization strategies might consider boosting with updated vaccines. Bivalent Omicron-containing BNT162b2 and mRNA1273.214 vaccines against Omicron BA.4 and BA.5 lineages have received regulatory approval^12,13^. Continued monitoring of the effects of SARS-CoV-2 mutations on disease severity and the effectiveness of therapeutics is warranted and COVID-19 vaccine policies should be based on evidence-based consensus.

## Methods

### Study population and general study design

This was a prospective cohort study of individuals over 18 years old from Rio de Janeiro, approved by the Brazilian National Council for Ethics in Research (CONEP) (protocol number 30639420.0.0000.5262), and all methods were performed in accordance with the Brazilian regulations (Resolution 441 of May 12^th^ 2011 from the Brazilian National Health Council). Individuals presenting to a public health clinic who tested positive for SARS-CoV-2 by real time RT-PCR and their household contacts were recruited beginning in May 2020^14^. We recruited and followed individuals who presented to the Germano Sinval Faria Health Center, which is a clinic located in the community of Manguinhos in Rio de Janeiro, Brazil. The clinic is part of Brazil’s public healthcare system and is responsible for an average of 30,000 primary care consultations per month. The community served by the clinic is characterized by high levels of social and economic deprivation.

Study visits consisted of a targeted physical exam, collection of nasopharyngeal swabs and 4 ml of blood for serological testing, and administration of a case report form (CRF). The CRF captured data on sociodemographic variables, comorbidities, exposure, risk factors, and clinical manifestations of COVID-19. Nasopharyngeal and oral swabs were tested by real-time reverse transcription polymerase chain reaction (RT-PCR) to amplify the E gene and the RdRp region of the Orf1ab gene of SARSCoV 2 using the SARS-CoV-2 Molecular E/RP Kit (Biomanguinhos, Rio de Janeiro, Brazil). Cycle thresholds (CTs) < 40 were considered positive. SARS-CoV-2 serology testing (IgG) was performed by using a chemiluminescence immunoassay targeting the nucleoprotein (N) gene and spike S1 subunit (Abbott Laboratories, Abbott Park, IL). All assays were performed according to the manufacturer’s instructions.

Following the rollout of COVID-19 immunization beginning in the first quarter of 2021, serum samples were consecutively collected from recipients of CoronaVac, ChAdOx1, and BNT162b2. Ad26.COV2.S vaccinees were excluded due to the small sample size. Serum samples were collected biweekly in the first month, and monthly until 6 months after complete vaccination. Sample collection schedule was restarted after the first and second booster. Participants immunized with ChAdOx1 and BNT162b2 were classified as vaccinated only (never infected) if they tested negative for anti-SARS-CoV-2 N IgG. Study participants were regularly tested for SARS-CoV-2 infection throughout their follow-up. The study included individuals that tested positive or negative for SARS-CoV-2 infection, before or after full vaccination, between March 2021 and August 2022.

### Virus neutralization testing

Titers of SARS-CoV-2 neutralizing antibodies were determined by a 90% plaque reduction neutralization test (PRNT_90_) using Vero cells (ATCC, CCL81) and a panel of reference isolates, including the VoCs Gamma P.1 (hCoV-19/Brazil/SC-FIOCRUZ-47330-1P/2021, EPI_ISL_6208350), Zeta P.2 (hCoV-19/Brazil/AL-FIOCRUZ-30270-1P/2020, EPI_ISL_2645635), Delta B.1.617.2 (hCoV-19/Brazil/MA-FIOCRUZ-25688-2P/2021, EPI_ISL_2645417), and Omicron BA.1 (hCoV-19/Brazil/SC-FIOCRUZ-63883-1P/2021, EPI_ISL_8430488), as previously described^15^. Briefly, heat-inactivated serum samples were tested in duplicates in serial twofold dilutions ranging from 1:10 to up to 1:320 for their ability to neutralize 50–80 plaque forming units (PFUs) in Vero cell monolayers in six-well plates by each one of the five infectious SARS-CoV-2 reference lineages isolates. After 48 h of incubation, plates were overlaid with neutral red solution, and after 72 h, PFUs were counted in a transilluminator. Virus neutralization was considered positive when a serum dilution of at least 1:10 reduced at least 90% of the PFUs of SARS-CoV-2. Geometric means were calculated to determine neutralizing antibodies titers for each SARS-CoV-2 lineage tested for further comparative analysis.

### Statistical analysis

Participants were stratified by vaccine, history of infection, and age (≤ 60 and > 60) and anti-spike IgG levels were evaluated to determine the magnitude and duration of anti-SARS-CoV-2 IgG response. The half-lives of anti-spike IgG levels were estimated using a linear decay model. To assess immune memory responses, we determined the fold-rise in the IgG geometric mean titer from 5 months after full vaccination (before boosting) to one month after the first booster dose. Differences in the neutralization titers between groups were evaluated with Kruskal–Wallis test. Results with *p*-value < 0.05 were considered significant.

### Ethics statement

The research protocol was approved by the Brazilian National Council for Ethics in Research (CONEP) (protocol number 30639420.0.0000.5262), and written informed consent was obtained from all participants.

## Supplementary Information

Supplementary Table S1.

## Data Availability

The datasets used and analyzed during the current study will be available on reasonable request from corresponding author.

## References

[1] Steele MK (2022). Estimated number of COVID-19 infections, hospitalizations, and deaths prevented among vaccinated persons in the US, December 2020 to September 2021. JAMA Netw. Open.

[2] Sobczak M, Pawliczak R (2022). COVID-19 vaccination efficacy in numbers including SARS-CoV-2 variants and age comparison: A meta-analysis of randomized clinical trials. Ann. Clin. Microbiol. Antimicrob..

[3] Centers for Disease Control and Prevention. SARS-CoV-2 Variant Classifications and Definitions. (2022).

[4] Gilboa M (2022). Durability of immune response after COVID-19 booster vaccination and association with COVID-19 omicron infection. JAMA Netw. Open.

[5] Wei J (2022). Antibody responses and correlates of protection in the general population after two doses of the ChAdOx1 or BNT162b2 vaccines. Nat. Med..

[6] Collie S, Champion J, Moultrie H, Bekker L-G, Gray G (2022). Effectiveness of BNT162b2 vaccine against omicron variant in South Africa. N. Engl. J. Med..

[7] Jassat W (2022). Clinical severity of COVID-19 in patients admitted to hospital during the omicron wave in South Africa: A retrospective observational study. Lancet Glob. Health.

[8] Abu-Raddad LJ, Chemaitelly H, Bertollini R (2021). Severity of SARS-CoV-2 reinfections as compared with primary infections. N. Engl. J. Med..

[9] Le Bert N (2020). SARS-CoV-2-specific T cell immunity in cases of COVID-19 and SARS, and uninfected controls. Nature.

[10] Dan JM (2021). Immunological memory to SARS-CoV-2 assessed for up to 8 months after infection. Science.

[11] Marchevsky NG (2022). An exploratory analysis of the response to ChAdOx1 nCoV-19 (AZD1222) vaccine in males and females. EBioMedicine.

[12] Chalkias S (2022). A bivalent omicron-containing booster vaccine against Covid-19. N. Engl. J. Med..

[13] U.S. Food and Drug Administration. Emergency Use Authorization (EUA) for an Unapproved Product Review Memorandum—Pfizer-BioNTech COVID-19 Vaccine, Bivalent (Original and Omicron BA.4/BA.5). (2022).

[14] Carvalho MS (2022). Incidence of SARS-CoV-2 over four epidemic waves in a low-resource community in Rio de Janeiro, Brazil: A prospective cohort study. Lancet Reg. Health Am..

[15] Pauvolid-Corrêa A (2022). Sera of patients infected by earlier lineages of SARS-CoV-2 are capable to neutralize later emerged variants of concern. Biol. Methods Protoc..

