# Supplementary material for: Reduced ability to neutralize the Omicron variant among adults after infection and complete vaccination with BNT162b2, ChAdOx1, or CoronaVac and heterologous boosting
Source: Sci Rep. 2023 May 8;13:7437. doi: 10.1038/s41598-023-34035-9 (PMC10165291; doi:10.1038/s41598-023-34035-9)
Supplement: Supplementary file 1 — Supplementary Table S1. [file 41598_2023_34035_MOESM1_ESM.docx]

**Table S1. Demographic characteristics and educational attainment of study participants**

| **Median age in years (IQR)** | All participants  N=524 | Second Booster  N=143 | *p*-value |
| --- | --- | --- | --- |
| **Median age in years (IQR)** | 44 (33-59) | 38 (31-56) | 0.15 |
| **Age (years)** | |  |  |
| ≤20, N(%) | 31 (5.9%) | 8 (5.6%) | 0.17 |
| 21-30, N(%) | 127 (24.2%) | 45 (31.5%) |  |
| 31-40, N(%) | 73 (13.9%) | 26 (18.2%) |  |
| 41-50, N(%) | 100 (19.1%) | 18 (12.6%) |  |
| 51-60, N(%) | 80 (15.3%) | 16 (11.2%) |  |
| Over 60 years of age, N (%) | 114 (21.8%) | 30 (21%) |  |
| **Female sex**, N(%) | 360 (68.7%) | 118 (82.5%) | 0.001 |
| **Education** | |  | <0.001 |
| Illiterate or did not graduate from primary school, N (%) | 69 (13.2%) | 29 (20.3%) |  |
| Graduated from primary school but did not graduate from secondary school, N(%) | 87(16.6%) | 38 (26.6%) |  |
| Graduated from secondary school but did not graduate from college, N(%) | 217 (41.4%) | 54 (37.8%) |  |
| Graduated from college, N (%) | 129 (24.6%) | 14 (9.8%) |  |
| Education missing, N (%) | 22 (4.2%) | 8 (5.6%) |  |
| **Race/ethnicity** | |  | 0.09 |
| Asian | 2 (0.4%) | 2 (1.4%) |  |
| Black | 97 (18.5%) | 35 (24.5%) |  |
| Multiracial | 172 (32.8%) | 53 (37.1%) |  |
| White | 232 (44.3%) | 47 (32.9%) |  |
| Ethnicity/race missing | 21 (4%) | 6 (4.2%) |  |
